# Psychometric properties of the Danish Hospital Anxiety and Depression Scale in patients with cardiac disease: results from the DenHeart survey

**DOI:** 10.1186/s12955-019-1264-0

**Published:** 2020-01-07

**Authors:** Anne Vinggaard Christensen, Jane K. Dixon, Knud Juel, Ola Ekholm, Trine Bernholdt Rasmussen, Britt Borregaard, Rikke Elmose Mols, Lars Thrysøe, Charlotte Brun Thorup, Selina Kikkenborg Berg

**Affiliations:** 1 Department of Cardiology, Rigshospitalet, Copenhagen University Hospital, Blegdamsvej 9, 2100 Copenhagen, Denmark; 2 Yale School of Nursing, Yale University, 400 West Campus Drive, Orange, CT 06477 USA; 3 National Institute of Public Health, University of Southern Denmark, Studiestræde 6, 1455 Copenhagen, Denmark; 4 Department of Cardiology, Herlev and Gentofte University Hospital, Kildegaardsvej 28, 2900 Hellerup, Denmark; 5 Cardiothoracic- and Vascular Department, Odense University Hospital, J.B.- Winslows Vej 4, 5000 Odense, Denmark; 6 Department of Cardiology, Aarhus University Hospital, Palle Juul-Jensens Blv. 99, 8200 Aarhus, Denmark; 7 Department of Cardiology, Odense University Hospital, University of Southern Denmark, J.B. Winsløws Vej 4, 5000 Odense, Denmark; 8 Department of Cardiology and Cardiothoracic Surgery, Clinical Nursing Research Unit, Aalborg University Hospital, Hobrovej 18-22, 9000 Aalborg, Denmark; 9 Institute of Clinical Medicine, University of Copenhagen, Blegdamsvej 3B, 2200 København N, Denmark

**Keywords:** Hospital anxiety and depression scale, Psychometric evaluation, Cardiac patients, Validity, Reliability

## Abstract

**Background:**

Anxiety and depression symptoms are common among cardiac patients. The Hospital Anxiety and Depression Scale (HADS) is frequently used to measure symptoms of anxiety and depression; however, no study on the validity and reliability of the scale in Danish cardiac patients has been done. The aim, therefore, was to evaluate the psychometric properties of HADS in a large sample of Danish patients with the four most common cardiac diagnoses: ischemic heart disease, arrhythmias, heart failure and heart valve disease.

**Methods:**

The DenHeart study was designed as a national cross-sectional survey including the HADS, SF-12 and HeartQoL and combined with data from national registers. Psychometric evaluation included analyses of floor and ceiling effects, structural validity using both exploratory and confirmatory factor analysis and hypotheses testing of convergent and divergent validity by relating the HADS scores to the SF-12 and HeartQoL. Internal consistency reliability was evaluated by Cronbach’s alpha, and differential item functioning by gender was examined using ordinal logistic regression.

**Results:**

A total of 12,806 patients (response rate 51%) answered the HADS. Exploratory factor analysis supported the original two-factor structure of the HADS, while confirmatory factor analysis supported a three-factor structure consisting of the original depression subscale and two anxiety subscales as suggested in a previous study. There were floor effects on all items and ceiling effect on item 8. The hypotheses regarding convergent validity were confirmed but those regarding divergent validity for HADS-D were not. Internal consistency was good with a Cronbach’s alpha of 0.87 for HADS-A and 0.82 for HADS-D. There were no indications of noticeable differential item functioning by gender for any items.

**Conclusions:**

The present study supported the evidence of convergent validity and high internal consistency for both HADS outcomes in a large sample of Danish patients with cardiac disease. There are, however, conflicting results regarding the factor structure of the scale consistent with previous research.

**Trial registration:**

ClinicalTrials.gov: NCT01926145.

## Background

Anxiety and depression symptoms are common among cardiac patients with prevalence rates of up to 30 and 20%, respectively, at hospital discharge and up to three months after hospitalization. This reflects the possible severity of the physical illness on other aspects of health [1, 2]. Previous studies have shown that anxiety and depression symptoms can predict future morbidity and mortality among cardiac patients [3, 4] underlining the importance of identifying these symptoms in order to initiate interventions to reduce them. A prerequisite for this is having a valid instrument to identify the symptoms.

The Hospital Anxiety and Depression Scale (HADS) was developed for patients with somatic illness admitted to the hospital [5] and is often used as a self-rating scale to screen for anxiety and depression symptoms across a wide range of patient and general populations. The scale includes two subscales, HADS-A and HADS-D measuring anxiety and depression symptoms, respectively. The scale is focused on the psychic symptoms of mood disorders, leaving out physical symptoms that can be confused with physical illness [5]. This is an advantage in cardiac populations where symptoms such as palpitations or dizziness might be related to the underlying cardiac disease and not a potential mood disorder.

HADS has been extensively tested for validity and reliability in English and other language versions, with satisfactory results across different patient populations, e.g. cardiac disease, cancer, psychological illness and in general populations [6–8]. Looking at previous validation studies of HADS in cardiac populations, however, there are differing results regarding the factor structure of the scale, Table 1. The originally proposed two-factor structure is confirmed in six studies [9–14], but eight studies find different versions of a three-factor structure to have the best fit depending on the analytic method used [12, 13, 15–20]. By contrast, one study finds a one-factor structure to have the best fit [21].

**Table 1:** Previous validations of HADS in patients with cardiac disease

Cronbach’s alpha								
Reference	Language	Population	Analytic methods	Number of factors	Sub scale content	HADS-A	HADS-D	Correlation between sub scales
Ayis et al. 2018 [9]	English	Stroke (*n* = 1443)	ML PCA CFA (and IRT)	2	Anxiety: 1,3,5,7,9,11,13 Depression: 2,4,6,8,10,12,14			
Kaur et al. 2015 [15]	Malaysian	Coronary artery disease (*n* = 189)	PCA CFA	3	Anxiety: 1,3,5,7,9,11,13 Anhedonia: 2,4,6,14 Psychomotor retardation: 8,10,12	0.89	0.69 Anhedonia: 0.70 Psychomotor retardation: 0.51	Anhedonia – psychomotor retardation: 0.35 Anhedonia – anxiety: 0.47 Psychomotor retardation – anxiety: 0.39
De Smedt et al. 2013 [10]	22 European countries	CABG, PCI, AMI, myocardial ischemia (*n* = 8745)	CFA	2	Anxiety: 1,3,5,7,9,11,13 Depression: 2,4,6,8,10,12,14	0.82	0.74	0.60
Cosco et al. 2012 [21]	English	Cardiovascular disease (*n* = 893)	MSA	1	1,2,3,4,5,6,7,9,10,11,12,13			
Emons et al. 2012 [16]	Dutch	Cardiac patients (*n* = 534)	MSA EFA CFA	3	Anxiety: 1,3,5,9,13 Depression: 2,4,6,8,10,12 Restlessness: 7,11,14			Depression – restlessness: 0.62 Restlessness – anxiety: 0.68 Depression – anxiety: 0.66
Kendel et al. 2010 [22]	German	CABG (*n* = 1271)	Rasch (HADS-D only)		Depression: 2,4,6,12			
Hunt-Shanks et al. 2010 [17]	English	Cardiac patients (*n* = 801)	CFA	3	Negative affect: 1,5,7,11 Autonomic anxiety: 3,9,13 Depression: 2,4,6,8,10,12,14			
Martin et al. 2008 [18]	German Chinese English	Coronary heart disease (*n* = 1793)	MGCFA	3	Antonomic anxiety: 3,9,13 Negative affectivity: 1,5,7,11Anhedonic depression: 2,4,6,8,10,12,14			
Pais-Ribeiro et al. 2007 [11]	Portuguese	Mixed patients incl. Coronary heart disease (*n* = 1322)	EFA CFA	2	Anxiety: 1,3,5,7,9,11,13 Depression: 2,4,6,8,10,12,14	0.76	0.81	0.58
Wang et al. 2006 [12]	Chinese	Coronary heart disease (*n* = 154)	CFA	2 or 3	2: Anxiety: 1,3,5,9,11 Depression: 2,4,6,7,8,10,12,14 3: Antonomic anxiety: 3,9,13 Negative affectivity: 1,5,7,11 Anhedonic depression: 2,4,6,8,10,12,14			
Barth and Martin 2005 [13]	German	Coronary heart disease (*n* = 1320)	EFA CFA	EFA: 2 CFA: 3	EFA: Anxiety: 1,3,5,7,9,11,13 Depression: 2,4,6,8,10,12,14 CFA: Psychomotor agitation: 1,7,11 Psychic anxiety: 3,5,9,13 Depression: 2,4,6,8,10,12,14			0.82 (between HADS-A and HADS-D)
Martin et al. 2004 [52]	Chinese	Acute coronary syndrome (*n* = 138)	CFA	3	Different models apply	0.79	0.55	
Martin et al. 2003 [19]	English	MI (*n* = 335)	CFA	3	Anhedonia: 2,4,6,8,10,12,14 Psychic anxiety: 3,5,9,13 Psychomotor agitation: 1,7,11	0.83–0.86 (3 timepoints)	0.76–0.80 (3 timepoints)	
Roberts et al. 2001 [14]	English	Female cardiac patients (*n* = 167)	CFA	2	Anxiety: 1,3,5,7,9,11,13 Depression: 2,4,6,8,10,12,14	0.85	0.80	0.60
Martin and Thompson 2000 [20]	English	MI (*n* = 194)	EFA	3	1: 2,4,6,7,8,10,12,14 2: 3,9,13 3: 1,5,11	0.76	0.72	0.54

*ML* maximum likelihood; *PCF* principal component analysis; *CFA* confirmatory factor analysis; *IRT* item response theory; *MSA* Mokken scale analysis; *EFA* exploratory factor analysis; *CABG* coronary artery bypass graft; *PCI* percutaneous coronary intervention; *AMI* acute myocardial infarction; *MGCFA* meta group confirmatory factor analysis; *MI* myocardial infarction

Differential item functioning (DIF) is a form of measurement error at item level by which patients from different groups with the same level of a construct being measured do not have the same scores. The presence of DIF by gender has been examined for HADS, but the results are not consistent [22–24].

HADS has been translated into Danish and is frequently used in clinical research but the psychometric properties of the Danish version have not been evaluated. Even though the scale has been found to be valid and reliable in previous studies, this is no assurance of equivalent validity when used in a different language, culture or context. Therefore, the aim of the current study was to evaluate the psychometric properties of the Danish HADS in a large population of patients with the most common cardiac diagnoses: ischemic heart disease, arrhythmias, heart failure and heart valve diseases.

## Methods

### Data collection and sample

Data was collected as part of the DenHeart study. The design and methods have been described in the pre-published protocol [25]. The DenHeart study was designed as a national cross-sectional survey combined with data from national registers at baseline and one year follow-up. Over a period of one year (April 2013–April 2014) all patients discharged or transferred from one of five national heart centers were asked to fill out a questionnaire at hospital discharge. Excluded were patients under the age of 18, patients without a Danish civil registration number, patients who did not understand Danish and patients who were unconscious when transferred from a heart center.

Based on their discharge diagnosis from the Danish National Patient Register [26], patients were divided into diagnostic sub-groups [2]. Included in the current analyses are patients with ischemic heart disease, arrhythmias, heart failure and heart valve diseases.

Furthermore, co-morbidity characteristics were collected from the Danish National Patient Register [26]. The Tu co-morbidity index was calculated including congestive heart failure, cardiogenic shock, arrhythmia, pulmonary oedema, malignancy, diabetes, cerebrovascular disease, acute/chronic renal failure and chronic obstructive pulmonary disease – all calculated ten years back [27].

Information on demographic characteristics were collected from the Civil Registration System [28] and the Danish Education Register [29].

### The HADS questionnaire

The HADS is a 14 item questionnaire originally developed to measure anxiety and depression symptoms in patients with somatic disease [5]. The instrument offers two subscales, HADS-A and HADS-D, each consisting of seven items and measuring anxiety and depression symptoms, respectively. HADS-A is focused on symptoms relating to generalized anxiety and HADS-D on symptoms relating to anhedonia, a central aspect of depression [30]. Each item is scored on a scale of 0–3 with each subscale score ranging from 0 to 21. Eight items are reverse scored with higher scores indicating a better response. These are reversed when summing the two subscales. The recommended cut-off values are 8–10 for possible presence of a mood disorder and ≥ 11 for probable presence of a mood disorder [5]. It has previously been found that among cardiac patients the minimal clinically important difference on the HADS is 1.7 points [31].

The Danish version of HADS has been frequently used for research purposes, both in observational studies and randomized controlled trials, as well as for screening purposes in clinical practice [2, 3, 32–36].

The translation of the HADS from English into Danish was evaluated by five independent assessors who were fluent in both English and Danish. For each item the equivalence of the translation was evaluated on a scale from 1 to 4, with higher numbers indicating stronger equivalence. The Translation Validity Index (TVI) was calculated as the proportion of assessments rated positively with score of 3 or 4 [37].

### Other instruments

The Short-Form 12 health survey (SF-12) is a brief, generic measure of health-related quality of life that generates both a physical (PCS) and a mental component score (MCS). Higher scores indicate better health status [16]. The SF-12 has been validated in a population of patients with coronary heart disease from 22 European countries with satisfactory results for construct validity and a Cronbach’s alpha of 0.87 for PCS and 0.84 for MCS, respectively, indicating high internal consistency reliability [10]. HeartQoL is a disease-specific questionnaire that measures quality of life in cardiac patients and produces a global score and two subscales: a physical and an emotional scale ranging from 0 to 3 with higher scores indicating better quality of life status [18–20]. The instrument has been validated in a large sample of coronary patients with results confirming both discriminative and convergent validity and high reliability with a Cronbach’s alpha of 0.87 for the emotional subscale and 0.91 for the physical one [38].

Furthermore, two single items on anxiety and depression allowed patients to rate anxiety and depression on a 10-point Likert scale.

### Psychometric properties of HADS

The following psychometric properties of the HADS were evaluated.

Floor and ceiling effects occur if more than 15% of the patients select the lowest or highest possible score on an item. Floor and ceiling effects can be an indication that extreme items are missing in either end of the scale, which can possibly limit its validity [39, 40].

Construct validity is defined as the degree to which an instrument measures what it is intended to measure. It is evaluated by testing hypotheses about an instrument – for example, relationships between parts of an instrument, relationships with scores of other instruments or differences between relevant groups [41]. An aspect of construct validity is structural validity, which is the degree to which the sub-scale scores of an instrument are an adequate reflection of the dimensions of the construct to be measured [41]. Structural validity was evaluated using exploratory factor analysis (EFA) and confirmatory factor analyses (CFA). CFA was conducted for the original two-factor structure suggested by Zigmond and Snaith [5], and also for four three-factor models [15, 42–44] and one one-factor model [21] found in previous studies including cardiac patients.

Construct validity was also examined through hypotheses testing by looking at HADS scores in relation to the MCS on SF-12, the emotional subscale of HeartQoL and a single item on anxiety and a single item on depression (convergent construct validity), and in relation to the PCS and physical subscale of HeartQoL (divergent construct validity).

We hypothesized high correlations (*r* > 0.60) between both HADS-A and HADS-D and the MCS score and the HeartQoL emotional score and high correlations between HADS-A and a single item measuring anxiety, and between HADS-D and a single item measuring depression. Furthermore, we hypothesized low correlations (*r* < 0.30) between HADS-A and HADS-D and PCS and HeartQoL physical as these measures were not supposed to be related to the HADS subscales.

Internal consistency reliability is an indicator of the extent to which the items of an instrument are internally correlated and therefore measure the same construct. This can be evaluated by calculating Cronbach’s alpha. A Cronbach’s alpha of between 0.70 and 0.95 is an indication of good internal consistency [40].

DIF is a form of measurement invariance at item level. DIF means that there are items for which patients from different groups with the same level of the construct being measured do not have the same scores. This can indicate that the item measures different things in the different groups. DIF can be uniform or non-uniform depending on whether the differences are present for all values of the scale or just for some values of the scale [45].

### Data analyses

Demographic and clinical characteristics are presented as frequencies or means with standard deviations (SD). Item score distributions are presented as means with SD, frequencies for each response category and missing data. Histograms and the Kolmogorov-Smirnov test were used to determine whether item scores deviated from the normal distribution.

Exploratory factor analysis was conducted using principal axis extraction based on eigenvalues greater than 1. Oblimin rotation was applied with a cut-off point of 0.30 as designating loading on a factor.

Confirmatory analyses were conducted with the weighted least squared means and variance (WLSMV) estimator. A Root Mean Square Error of Approximation (RMSEA) estimate below 0.06 along with Comparative Fit Index (CFI) and Tucker Lewis Index (TLI) estimates above 0.95 indicated a good model fit [46].

Both the EFA and the CFA were conducted on the total population. Extensive previous literature exists that provide suggestions for models to be tested in the CFA.

Spearman’s rank-order correlations were used to determine convergent and divergent validity as data were not normally distributed. Convergent validity between HADS, SF-12 and HeartQoL subscales was examined by stratifying mean scores of MCS, PCS, and HeartQoL emotional and HeartQoL physical by HADS-A and HADS-D scores above and below 8.

Internal consistency was evaluated by calculating Cronbach’s alpha for subscales and also by corrected item-total correlations.

DIF was examined using multivariate ordinal logistic regression with items as the dependent variable and gender and total score (HADS-A or HADS-D depending on the item) as the independent variables. Because the proportional odds assumption was not fulfilled a partial proportional odds model was used. DIF was evaluated by different criteria. Uniform DIF can be considered if the odds ratio (OR) for gender is statistically significantly different from 1 [45]. Interactions between gender and total score were included to evaluate possible non-uniform DIF. A statistically significant interaction can be an indication of non-uniform DIF [45]. Because of the large sample size and the risk of finding statistically significant results with no or very little clinical meaning, DIF was also evaluated by Nagelkerke’s R.^2^ A difference in R^2^ of more than 0.03 between models was an indication of noticeable DIF (both uniform and non-uniform) [45].

Only patients with complete responses to the HADS were included in the analyses.

Analyses were conducted using SAS version 9.4, IBM SPSS version 25 and Mplus version 7.4.

## Results

### Demographic and clinical profile

Out of 25,241 eligible patients, 12,806 had complete responses to the HADS questionnaire giving a response rate of 51%. Demographic and clinical characteristics are presented in Table 2.

**Table 2:** Demographic and clinical characteristics

n	12,806
Male, n (%)	8953 (69.9)
Age, mean (SD)	65.1 (12.1)
Marital status (n,%)	
Married Divorced Widowed Unmarried	8307 (64.9) 1728 (13.5) 1533 (12.0) 1238 (9.6)
Educational level (n,%)	
Basic school Upper secondary or vocational school Higher education Missing	3903 (30.5) 5595 (43.7) 3018 (23.5) 290 (2.3)
Cardiac diagnosis (n,%)	
Ischemic heart disease Arrhythmias Heart failure Heart valve diseases	6832 (53.3) 4121 (32.2) 917 (7.2) 936 (7.3)
Co-morbidity (n,%)	
Hypertension	4424 (34.6)
Ventricular arrhythmia	589 (4.6)
Ischemic heart disease	5544 (43.3)
Myocardial infarction	2408 (18.8)
Diabetes	1257 (9.8)
Heart failure	2210 (17.3)
Renal disease	426 (3.3)
Chronic obstructive pulmonary disease	837 (6.5)
Tu comorbidity score (n,%)	
0	5271 (41.2)
1	4378 (34.2)
2	2062 (16.1)
≥3	1095 (8.5)

### Item score statistics and translation validity index

The item score statistics are presented in Table 3. Item 8 showed markedly different scores compared to the rest of the items, with more patients using high response categories, Table 3. There were floor effects on all items and a ceiling effect on item 8, Table 3.

**Table 3:** Item and score statistics

		Score distribution, n (%)				
	Mean (SD)	0	1	2	3	Missing
HADS-A n = 12,806	5.79 (4.19)					
1. I feel tense or ‘wound up’*	1.05 (0.83)	3471 (25.8)	6413 (47.6)	2662 (19.8)	745 (5.5)	172 (1.3)
3. I get a sort of frightened feeling as if something awful is about to happen*	1.09 (0.90)	4050 (30.1)	4702 (34.9)	3754 (27.9)	361 (5.7)	196 (1.5)
5. Worrying thoughts go through my mind*	0.90 (0.89)	5189 (38.5)	5027 (37.3)	2244 (16.7)	790 (5.9)	213 (1.6)
7. I can sit at ease and feel relaxed	0.73 (0.73)	5673 (42.1)	5746 (42.7)	1721 (12.8)	156 (1.2)	167 (1.2)
9. I get a sort of frightened feeling like ‘butterflies’ in the stomach	0.62 (0.72)	6596 (50.0)	5354 (39.8)	1009 (7.5)	304 (2.3)	200 (1.5)
11. I feel restless as I have to be on the move*	0.88 (0.81)	4874 (36.2)	5549 (41.2)	2444 (18.2)	413 (3.1)	183 (1.4)
13. I get sudden feelings of panic*	0.52 (0.69)	7691 (57.1)	4355 (32.4)	1035 (7.7)	163 (1.2)	219 (1.6)
HADS-D n = 12,806	4.29 (3.65)					
2. I still enjoy the things I used to enjoy	0.72 (0.78)	6080 (41.2)	5332 (39.6)	1428 (10.6)	433 (3.2)	190 (1.4)
4. I can laugh and see the funny side of things	0.37 (0.64)	9403 (69.8)	2991 (22.2)	766 (5.7)	131 (1.0)	172 (1.3)
6. I feel cheerful*	0.51 (0.65)	8309 (61.7)	3358 (24.9)	1417 (10.5)	209 (1.6)	170 (1.3)
8. I feel as if I am slowed down*	1.40 (0.93)	2078 (15.4)	5912 (43.9)	3177 (23.6)	2122 (15.8)	174 (1.3)
10. I have lost interest in my appearance*	0.43 (0.69)	8983 (66.7)	3076 (22.9)	1080 (8.0)	138 (1.0)	186 (1.4)
12. I look forward with enjoyment to things	0.52 (0.74)	8119 (60.3)	3593 (26.7)	1313 (9.8)	225 (1.7)	213 (1.6)
14. I can enjoy a good book or radio or TV program	0.37 (0.69)	9654 (71.7)	2608 (19.4)	705 (5.2)	289 (2.2)	207 (1.5)

Each item is scored on a scale of 0–3 with each subscale ranging from 0 to 21. For six items higher scores indicate a worse response. The eight items highlighted with * are reverse scored. These are reversed when summing the subscales

Of the 14 items, 12 had an TVI of 100%, and two (items 3 and 11) had TVI of 60% (both of these were a part of HADS-A. The TVI for the total scale was 94%, Additional file 1: Table S1.

### Factor structure

The results from the EFA indicate that the original two-factor structure of the HADS seems to fit in this cardiac population. However, item 7 showed almost the same loading on each subscale, Table 4. The correlation between HADS-A and HADS-D was 0.66.

**Table 4:** Exploratory factor analysis - rotated factor matrix^a^

	Factor	
	1	2
Item 9. I get a sort of frightened feeling like ‘butterflies’ in the stomach	0.81	
Item 3. I get a sort of frightened feeling as if something awful is about to happen	0.80	
Item 5. Worrying thoughts go through my mind	0.69	
Item 13. I get sudden feelings of panic	0.71	
Item 1. I feel tense or ‘wound up’	0.60	
Item 7. I can sit at ease and feel relaxed	0.41	0.36
Item 11. I feel restless as I have to be on the move	0.46	
Item 12. I look forward with enjoyment to things		0.79
Item 6. I feel cheerful		0.67
Item 2. I still enjoy the things I used to enjoy		0.72
Item 4. I can laugh and see the funny side of things		0.62
Item 8. I feel as if I am slowed down		0.54
Item 10. I have lost interest in my appearance		0.55
Item 14. I can enjoy a good book or radio or TV program		0.45
Cumulative % of variance explained	45.22	53.99
Eigenvalue	6.33	1.23

^a^Exploratory factor analyses using principal axis extraction based in eigenvalues

greater than 1, Oblimin rotation and cut-off point of 0.30

Loadings> 0.40 in bold

**Fig. 1:**
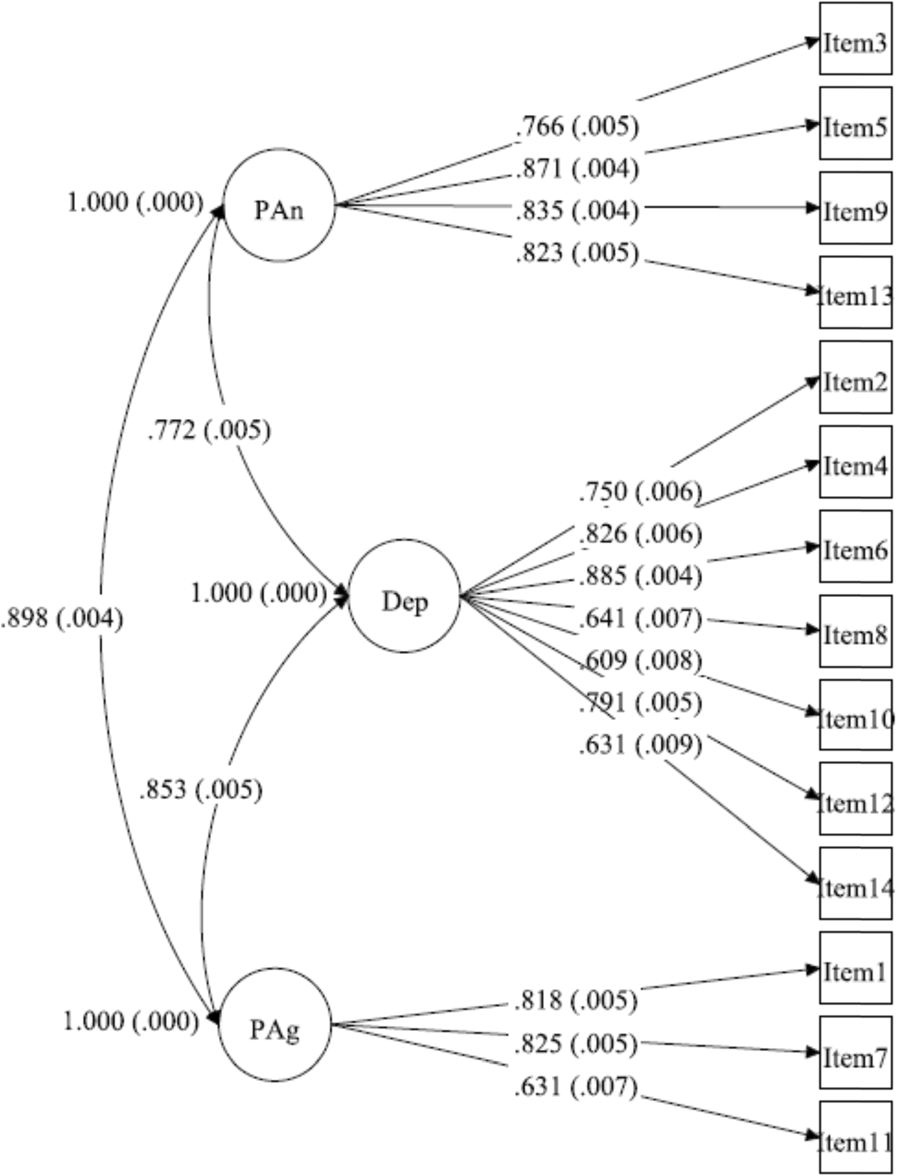
Diagram from the confirmatory factor analysis presenting the model with the best fit. Standardized loadings (SE). PAn = psychic anxiety; Dep = depression; PAg = psychomotor agitation

The CFA indicated that the three-factor structure suggested by Friedman et al. [44] showed the best fit for the models tested, Table 5. The diagram from the CFA of the three-factor structure suggested by Friedman et al. [44] is presented in Fig. 1.

**Table 5:** Fit indices for confirmatory factor analyses of factor structures proposed in previous studies

			RMSEA				
Models	Number of factors	Sub scale content	RMSEA	90% CI	p-value	CFI	TLI
Zigmond and Snaith 1983 [5]	2	HADS-A: 1,3,5,7,9,11,13 HADS-D: 2,4,6,8,10,12,14	0.071	0.069;0.072	< 0.001	0.973	0.968
Dunbar et al. 2000 [43]	3	Negative affect: 1,5,7,11 Autonomic anxiety: 3,9,13 Depression: 2,4,6,8,10,12,14	0.061	0.059;0.062	< 0.001	0.981	0.976
Friedman et al. 2001 [44]	3	Psychomotor agitation: 1,7,11 Psychic anxiety: 3,5,9,13 Depression: 2,4,6,8,10,12,14	0.060	0.058;0.061	< 0.001	0.981	0.977
Caci et al. 2003 [42]	3	Anxiety: 1,3,5,9,13 Depression: 2,4,6,8,10,12 Restlessness: 7,11,14	0.064	0.062;0.065	< 0.001	0.979	0.974
Kaur et al. 2015 [15]	3	Anxiety: 1,3,5,7,9,11,13 Anhedonia: 2,4,6,14 Psychomotor retardation: 8,10,12	0.069	0.068;0.071	< 0.001	0.975	0.969
Cosco et al. 2012 [21]	1	1,2,3,4,5,6,7,9,10,11,12,13	0.111	0.109;0.113	< 0.001	0.945	0.932

### Convergent and divergent validity

Looking at MCS, PCS, HeartQoL emotional and HeartQoL physical scores in relation to HADS scores, patients with scores below 8 on both HADS-A or HADS-D had high scores on MCS and HeartQoL emotional. Conversely, patients with HADS-A and HADS-D scores above 8 have the lowest scores. The same pattern is found in PCS and HeartQoL physical scores, Table 6.

**Table 6:** HADS scores in relation to SF-12 and HeartQoL scores

			HADS-A	
			< 8	≥8
HADS-D		n (%)	8211 (64.1)	553 (4.3)
	< 8	MCS, mean (SD)	53.03 (7.96)	42.96 (8.63)
		PCS, mean (SD)	44.13 (10.43)	34.11 (9.07)
		HeartQoL emotional, mean (SD)	2.50 (0.56)	1.56 (0.68)
		HeartQoL physical, mean (SD)	1.83 (0.83)	1.47 (0.81)
		n (%)	2147 (16.8)	1895 (14.8)
	≥8	MCS, mean (SD)	42.01 (9.16)	34.11 (9.07)
		PCS, mean (SD)	33.44 (9.93)	35.53 (10.03)
		HeartQoL emotional, mean (SD)	1.92 (0.70)	1.04 (0.69)
		HeartQoL physical, mean (SD)	1.02 (0.71)	0.95 (0.70)

HADS Hospital Anxiety and Depression Scale; SF-12 = Short Form 12; HADS-A = Hospital Anxiety and Depression Scale – Anxiety; HADS-D = Hospital Anxiety and Depression Scale – Depression; MCS = Mental Component Scale; PCS = Physical Component Scale; SD = Standard deviation

The cut-off of 8 is used as an indicator of possible mood disorder

Correlations between HADS-A and MCS and HeartQoL emotional were 0.67 and 0.75, respectively. Correlations between HADS-D and MCS and HeartQoL emotional were 0.66 and 0.63, respectively. The correlation between HADS-A and the single item on anxiety was 0.68 and between HADS-D and the single item on depression it was 0.59. This confirmed the stated hypotheses about convergent validity. However, the two single items were highly correlated (0.76).

Correlations between HADS-A and PCS and HeartQoL physical were 0.25 and 0.35, respectively. Correlations between HADS-D and PCS and HeartQoL physical were 0.50 and 0.55, respectively. This did not confirm the hypotheses on divergent validity for HADS-D.

### Internal consistency

For HADS-A mean inter-item correlation was 0.50 (range 0.35–0.61) and Cronbach’s alpha was 0.87. The corrected item-total correlations ranged from 0.52 to 0.71. Cronbach’s alpha would not be improved by the deletion of any item.

For HADS-D mean inter-item correlation was 0.41 (range 0.24–0.58). Cronbach’s alpha was 0.82. The corrected item-total correlations ranged from 0.44 to 0.67. Cronbach’s alpha would not be improved by the deletion of any item.

For all HADS items the mean inter-item correlation was 0.40 (range 0.24–0.61).

Looking at the three-factor structure, the Cronbach’s alpha for the psychomotor agitation subscale was 0.74 and 0.83 for the psychic anxiety subscale. The HADS-D subscale was unchanged with a Cronbach’s alpha of 0.82. Cronbach’s alpha would not be improved by the deletion of any item.

### Differential item functioning

There were indications of DIF for item 3, 4 and 13 where women were more likely to have high item scores compared to men and for items 11 and 14 where men were more likely to have high item scores compared to women. There were significant interactions between item and subscale for items 1, 2, 5, 7, 8, 9 and 12, which is an indication of non-uniform DIF. However, in analysis using Nagelkerke’s R^2^ there was no noticeable DIF for any item, Table 7.

**Table 7:** Differential item functioning tested for gender

	OR (95% CI)^a^ for item responses 1, 2 and 3	Overall p-value	Significant interaction between gender and sub scale	Nagelkerke’s R^2^ Step 1	Nagelkerke’s R^2^ Step 2	Nagelkerke’s R^2^ Step 3	DIF R^2 b^
HADS-A							
Item 1. I feel tense or ‘wound up’			X	0.6712	0.6714	0.6717	0.0005
Item 3. I get a sort of frightened feeling as if something awful is about to happen	1: 0.948 (0.782;1.149) 2: 0.801 (0.719;0.892) 3: 0.916 (0.820;1.023)	0.0007		0.6287	0.6293	0.6295	0.0008
Item 5. Worrying thoughts go through my mind			X	0.6924	0.6930	0.6932	0.0008
Item 7. I can sit at ease and feel relaxed			X	0.6008	0.6011	0.6018	0.0010
Item 9. I get a sort of frightened feeling like ‘butterflies’ in the stomach			X	0.6718	0.6726	0.6730	0.0012
Item 11. I feel restless as I have to be on the move	1:1.670 (1.315;2.122) 2: 1.385 (1.240;1.545) 3: 1.423 (1.289;1.571)	<.0001		0.4746	0.4785	0.4788	0.0042
Item 13. I get sudden feelings of panic	1: 0.667 (0.462;0.963) 2: 0.712 (0.600;0.845) 3: 0.781 (0.703;0.868)	<.0001		0.6291	0.6307	0.6308	0.0017
**HADS-D**							
Item 2. I still enjoy the things I used to enjoy			X	0.6107	0.6112	0.6116	0.0009
Item 4. I can laugh and see the funny side of things	1: 0.865 (0.587;1.274) 2: 0.917 (0.765;1.099) 3: 0.805 (0.719;0.902)	0.0025		0.5739	0.5747	0.5747	0.0008
Item 6. I feel cheerful	1: 1.079 (0.750;1.551) 2: 1.089 (0.937;1.266) 3: 1.071 (0.956;1.200)	0.5055		0.6381	0.6381	0.6385	0.0004
Item 8. I feel as if I am slowed down			X	0.5660	0.5676	0.5684	0.0024
Item 10. I have lost interest in my appearance	1: 0.813 (0.561;1.177) 2: 1.050 (0.905;1.218) 3: 0.956 (0.866;1.054)	0.3345		0.4235	0.4237	0.4239	0.0003
Item 12. I look forward with enjoyment to things			X	0.6136	0.6142	0.6143	0.0007
Item 14. I can enjoy a good book or radio or TV program	1: 2.132 (1.558;2.918) 2: 1.612 (1.361;1.909) 3: 1.431 (1.289;1.587)	<.0001		0.3805	0.3853	0.3855	0.0050

^a^ Partial proportional odds model with item response as dependent variable and gender and subscale as independent variable. Men are reference

Step 1: Partial proportional odds model with item response as dependent variable including subscale

Step 2: Partial proportional odds model with item response as dependent variable including subscale and gender

Step 2: Partial proportional odds model with item response as dependent variable including subscale, gender and an interaction between the two

^b^ Indication of both uniform and non-uniform DIF

## Discussion

In the present study the psychometric properties of the HADS in a large sample of Danish cardiac patients were evaluated. Floor effects were found on all items and ceiling effect on item 8. The original two-factor structure of the scale was confirmed in EFA, but CFA indicated a three-factor structure. The hypotheses proposed were supported for both subscales, providing evidence for convergent validity. However, for HADS-D the hypotheses proposed for divergent validity were not supported. Thus, divergent validity is not indicated. Internal consistency was good for both HADS-A and HADS-D.

The factor analyses indicate that the factor structure of the HADS is not completely clear. The EFA confirmed the original two-factor structure suggested by Zigmond and Snaith [5], but the CFA showed that the three-factor structure as found by Friedman et al. [44] in a French sample of patients suffering from major depression had the best model fit. The same result was found by Barth and Martin in a German coronary heart disease population [13]. Several other studies have found variations of a three-factor structure to have the best model fit for the HADS as indicated in Table 5. The differences in factor structure found across studies might be explained by different methodology such as data extraction method, model fit criteria, translation or type of patients included.

When considering the content of the three factors suggested by Friedman et al. [44]; psychomotor agitation (item 1, 7, 11), psychic anxiety (item 3, 5, 9, 13) and depression (item 2, 4, 6, 8, 10, 12, 14), the division of items from the original HADS-A into two factors can make sense as relating to two different dimensions of anxiety disorder. The items in the psychomotor agitation subscale relate to physical feelings of restlessness and agitation while the items in the psychic anxiety subscale relate to emotional representation of anxiety with worrying and nervous thoughts. Agitation is, however, also a common symptom among patients with depressive disorders and can occur as a side effect of antidepressant medication [47].

The interrelatedness between symptoms of anxiety and depression is further evident in the high correlations between HADS-A and HADS-D. This did not change when looking at the three-factor structure instead. It has previously been argued that a high correlation between anxiety and depression is to be expected, not because of common symptoms but because it is possible that anxiety can lead to depression and that depression can lead to anxiety. It is also possible that the two disorders result from a common cause. The causality of this relationship cannot, however, be determined from cross-sectional data [48].

In the EFA item 7 was found to load almost equally on both factors. This has been found in previous studies as well [13]. Item 7 reads ‘I can sit at ease and feel relaxed’; this may reflect aspects of both anxiety and depression.

Eight items in the HADS are reversely scored. This is a recommended method to avoid acquiescence bias which is the tendency for respondents of a survey to agree with statements regardless of their content. However, research suggests that individual differences in response styles can systematically affect the factor structure [49]. The uncertainty of the factor structure of the HADS is not necessarily a reason to discard the instrument, but rather to be clear on the purpose of using the scale. The two-factor structure may prove useful as a simple indication of either anxiety or depression. The possible presence of a third factor indicates that the scale may provide more refined results regarding different aspects of anxiety, rather than just an indication of generalized anxiety. Because the results regarding factor structure were not clear, the two-factor structure originally proposed was used in the remaining analyses for the paper.

There were floor effects on all items, which may indicate that the number of extreme response categories is not sufficient. As the HADS was developed to detect indications of a mood disorder, which is not present in the majority of the population, even a population with severe illness, it is not surprising that there are floor effects. Item 8 also showed a ceiling effect. The item reads ‘I feel as if I am slowed down’. In a population of elderly, severely ill patients just discharged, it is not surprising that this feeling would be prevalent. This item is susceptible to influence from either age or disease which is a bias in terms of validity as an indicator of mood.

The analyses of DIF indicated that there could be potential problems with DIF for several items. However, because of the risk of finding statistically significant results of minimal clinical importance in this large population, changes in Nagelkerke’s R^2^ between models were given priority. These indicated no noticeable DIF for any items. The presence of DIF for gender has been explored in previous studies [22–24, 50], but only one study found substantial DIF for item 14, with men being more likely to endorse this item [22].

When considering the usefulness of the HADS in clinical practice it should also be noted that HADS has been shown to predict morbidity and mortality in this patient population and similar patient populations [3, 4, 51].

### Limitations of the study

There is no description of the process of how the HADS was translated into Danish from the questionnaire owner, so it is not clear whether the translation has followed the recommended steps to ensure cross-cultural validity [45]. The current analyses are, in fact, the first specific investigation of the psychometric properties of the Danish language version of HADS. For the current study, we evaluated the TVI for each item and the total scale with satisfactory results. Items 3 and 11 (both in HADS-A) received the lowest rating (60%).

Newer methods for exploring internal consistency exist, e.g. the use of McDonalds omega. However, for consistency with the methods chosen throughout this paper and for comparison with other HADS validation studies we chose to include Cronbach’s alpha.

The large sample size in this study is an advantage because of statistical power and because it allows a heterogeneous sample. There is, however, a risk of finding statistically significant results of minimal clinical importance. Therefore, we have not only looked at *p*-values to determine validity, but rather measures of strength of correlation, internal consistency and Nagelkerke’s R^2^ for analyses of DIF.

The response rate was 51%, which is to be expected in a population of severely ill patients on the day of hospital discharge. This may raise concerns about representativeness, however, the proportions of patients in the diagnostic sub-groups were similar to that of the entire eligible population, and responders and non-responders were comparable in terms of their demographic and clinical profiles, suggesting a representative sample [2]. We did, however, find a higher mortality rate in non-responders compared to responders [4].

In the present study we used a single question on anxiety and depression to measure convergent validity. However, the two questions were highly correlated. Including more comprehensive instruments to measure anxiety and depression would have been optimal to examine convergent validity. These were, however, not available in the data.

## Conclusions

The findings of this study supported the validity and reliability of the HADS in a sample of Danish patients with cardiac disease. EFA supported the original two-factor structure of the scale, while CFA supported a three-factor structure consisting of the original depression subscale and two anxiety subscales; psychomotor agitation and psychic anxiety. The hypotheses regarding convergent validity were confirmed, but those regarding divergent validity were not confirmed for HADS-D. Internal consistency was good with a Cronbach’s alpha of 0.87 for HADS-A and 0.82 for HADS-D. There were no indications of noticeable DIF by gender for any items.

## Supplementary information

**Additional file 1: Table S1.** Translation Validity Index (TVI) for the Danish translation of Hospital Anxiety and Depression Scale (HADS)

## Data Availability

Danish legislation on data security prohibits sharing of data.

## Abbreviations

CFA: Confirmatory factor analysis
CFI: Comparative Fit Index
DIF: Differential item functioning
EFA: Exploratory factor analysis
HADS: Hospital Anxiety and Depression Scale
MCS: Mental component score
OR: Odds ratio
PCS: Physical component score
RMSEA: Root Mean Square Error of Approximation
SD: Standard deviation
SF-12: Short-Form 12
TLI: Tucker Lewis Index
WLSMV: Weighted least squared means and variance

## References

[1] Moser DK, Dracup K, Evangelista LS, Zambroski CH, Lennie TA, Chung ML (2010). Comparison of prevalence of symptoms of depression, anxiety, and hostility in elderly patients with heart failure, myocardial infarction, and a coronary artery bypass graft. Heart Lung.

[2] Berg SK, Rasmussen TB, Thrysoee L, Lauberg A, Borregaard B, Christensen AV (2017). DenHeart: differences in physical and mental health across cardiac diagnoses at hospital discharge. J Psychosom Res.

[3] Berg SK, Thygesen LC, Svendsen JH, Christensen AV, Zwisler AD (2014). Anxiety predicts mortality in ICD patients: results from the cross-sectional National Copenheart Survey with register follow-up. Pacing Clin Electrophysiol.

[4] Berg SK, Thorup CB, Borregaard B, Christensen AV, Thrysoee L, Rasmussen TB (2019). Patient-reported outcomes are independent predictors of one-year mortality and cardiac events across cardiac diagnoses: findings from the national DenHeart survey. Eur J Prev Cardiol.

[5] Zigmond AS, Snaith RP (1983). The hospital anxiety and depression scale. Acta Psychiatr Scand.

[6] Herrmann C (1997). International experiences with the hospital anxiety and depression scale - a review of validation data and clinical results. J Psychosom Res.

[7] Bjelland I, Dahl AA, Haug TT, Neckelmann D (2002). The validity of the hospital anxiety and depression scale. An updated literature review. J Psychosom Res.

[8] Cosco TD, Doyle F, Ward M, McGee H (2012). Latent structure of the hospital anxiety and depression scale: a 10-year systematic review. J Psychosom Res.

[9] Ayis SA, Ayerbe L, Ashworth M, DA Wolfe C (2018). Evaluation of the hospital anxiety and depression scale (HADS) in screening stroke patients for symptoms: item response theory (IRT) analysis. J Affect Disord.

[10] De Smedt D, Clays E, Doyle F, Kotseva K, Prugger C, Pajak A (2013). Validity and reliability of three commonly used quality of life measures in a large European population of coronary heart disease patients. Int J Cardiol.

[11] Pais-Ribeiro J, Silva I, Ferreira T, Martins A, Meneses R, Baltar M (2007). Validation study of a Portuguese version of the hospital anxiety and depression scale. Psychol Health Med.

[12] Wang W, Lopez V, Martin CR (2006). Structural ambiguity of the Chinese version of the hospital anxiety and depression scale in patients with coronary heart disease. Health Qual Life Outcomes.

[13] Barth J, Martin CR (2005). Factor structure of the hospital anxiety and depression scale (HADS) in German coronary heart disease patients. Health Qual Life Outcomes.

[14] Roberts SB, Bonnici DM, Mackinnon AJ, Worcester MC (2001). Psychometric evaluation of the hospital anxiety and depression scale (HADS) among female cardiac patients. Br J Health Psychol.

[15] Kaur S, Zainal NZ, Low WY, Ramasamy R, Sidhu JS (2015). Factor structure of hospital anxiety and depression scale in Malaysian patients with coronary artery disease. Asia Pacific J Public Heal.

[16] Emons WHM, Sijtsma K, Pedersen SS (2012). Dimensionality of the hospital anxiety and depression scale (HADS) in cardiac patients. Assessment.

[17] Hunt-Shanks T, Blanchard C, Reid R, Fortier M, Cappelli M (2010). A psychometric evaluation of the hospital anxiety and depression scale in cardiac patients: addressing factor structure and gender invariance. Br J Health Psychol.

[18] Martin CR, Thompson DR, Barth J (2008). Factor structure of the hospital anxiety and depression scale in coronary heart disease patients in three countries. J Eval Clin Pract.

[19] Martin CR, Lewin RJP, Thompson DR (2003). A confirmatory factor analysis of the hospital anxiety and depression scale in coronary care patients following acute myocardial infarction. Psychiatry Res.

[20] Martin CR, Thompson DR (2000). A psychometric evaluation of the hospital anxiety and depression scale in coronary care patients following acute myocardial infarction. Psychol Health Med.

[21] Cosco TD, Doyle F, Watson R, Ward M, McGee H (2012). Mokken scaling analysis of the hospital anxiety and depression scale in individuals with cardiovascular disease. Gen Hosp Psychiatry.

[22] Kendel F, Wirtz M, Dunkel A, Lehmkuhl E, Hetzer R, Regitz-Zagrosek V (2010). Screening for depression: Rasch analysis of the dimensional structure of the PHQ-9 and the HADS-D. J Affect Disord.

[23] Djukanovic I, Carlsson J, Årestedt K (2017). Is the hospital anxiety and depression scale (HADS) a valid measure in a general population 65–80 years old?. A psychometric evaluation study Health Qual Life Outcomes.

[24] Cameron IM, Crawford JR, Lawton K, Reid IC (2013). Differential item functioning of the HADS and PHQ-9: an investigation of age, gender and educational background in a clinical UK primary care sample. J Affect Disord.

[25] Berg SK, Svanholm J, Lauberg A, Borregaard B, Herning M, Mygind A (2014). Patient-reported outcomes at hospital discharge from heart Centres, a national cross-sectional survey with a register-based follow-up: the DenHeart study protocol. BMJ Open.

[26] Lynge E, Sandegaard JL, Rebolj M (2011). The Danish National Patient Register. Scand J Public Health.

[27] Tu JV, Austin PC, Walld R, Roos L, Agras J, McDonald KM (2001). Development and validation of the Ontario acute myocardial infarction mortality prediction rules. J Am Coll Cardiol.

[28] Pedersen CB (2011). The Danish civil registration system. Scand J Public Health..

[29] Jensen VM, Rasmussen AW (2011). Danish education registers. Scand J Public Health..

[30] Snaith RP (2003). The hospital depression and anxiety scale. Health Qual Life Outcomes.

[31] Lemay KR (2018). Tulloch HE.

[32] Hojskov IE, Moons P, Hansen NV, Greve H, Olsen DB, Cour SL (2016). Early physical training and psycho-educational intervention for patients undergoing coronary artery bypass grafting. The SheppHeart randomized 2 x 2 factorial clinical pilot trial. Eur J Cardiovasc Nurs.

[33] Sibilitz KL, Berg SK, Thygesen LC, Hansen TB, Kober L, Hassager C (2015). High readmission rate after heart valve surgery: a nationwide cohort study. Int J Cardiol.

[34] Rasmussen TB, Zwisler AD, Thygesen LC, Bundgaard H, Moons P, Berg SK (2017). High readmission rates and mental distress after infective endocarditis - results from the national population-based CopenHeart IE survey. Int J Cardiol.

[35] Berg SK, Herning M, Svendsen JH, Christensen AV, Thygesen LC (2016). The Screen-ICD trial. Screening for anxiety and cognitive therapy intervention for patients with implanted cardioverter defibrillator (ICD): a randomised controlled trial protocol. BMJ Open.

[36] Berg SK, Herning M, Thygesen LC, Cromhout PF, Wagner MK, Nielsen KM (2019). Do patients with ICD who report anxiety symptoms on hospital anxiety and depression scale suffer from anxiety?. J Psychosom Res.

[37] Tang S, Dixon J (2002). Instrument translation and evaluation of equivalence and psychometric properties: the Chinese sense of coherence scale. J Nurs Meas.

[38] De Smedt D, Clays E, Hofer S, Oldridge N, Kotseva K, Maggioni AP (2016). Validity and reliability of the HeartQoL questionnaire in a large sample of stable coronary patients: the EUROASPIRE IV study of the European Society of Cardiology. Eur J Prev Cardiol.

[39] McHorney CA, Tarlov AR (1995). Individual-patient monitoring in clinical practice: are available health status surveys adequate?. Qual Life Res.

[40] Terwee CB, Bot SD, de Boer MR, van der Windt DA, Knol DL, Dekker J (2007). Quality criteria were proposed for measurement properties of health status questionnaires. J Clin Epidemiol.

[41] Mokkink LB, Terwee CB, Patrick DL, Alonso J, Stratford PW, Knol DL (2010). The COSMIN study reached international consensus on taxonomy, terminology, and definitions of measurement properties for health-related patient-reported outcomes. J Clin Epidemiol.

[42] Caci H, Baylé FJ, Mattei V, Dossios C, Robert P, Boyer P (2003). How does the hospital and anxiety and depression scale measure anxiety and depression in healthy subjects?. Psychiatry Res.

[43] Dunbar M, Ford G, Hunt K, Der G (2000). A confirmatory factor analysis of the hospital anxiety and depression scale: comparing empirically and theoretically derived structures. Br J Clin Psychol.

[44] Friedman S, Samuelian JC, Lancrenon S, Even C, Chiarelli P (2001). Three-dimensional structure of the hospital anxiety and depression scale in a large French primary care population suffering from major depression. Psychiatry Res.

[45] de Vet HC, Terwee CB, Mokkink LB, Knol DL (2011). Measurement in medicine: a practical guide.

[46] Hu L, Bentler PM (1999). Cutoff criteria for fit indexes in covariance structure analysis: conventional criteria versus new alternatives. Struct Equ Model A Multidiscip J.

[47] Nutt DJ (1999). Care of depressed patients with anxiety symptoms. J Clin Psychiatry.

[48] Burns DD, Eidelson RJ (1998). Why are depression and anxiety correlated? A test of the tripartite model. J Consult Clin Psychol.

[49] Hidalgo-Rasmussen C, González-Betanzos F (2019). The treatment of acquiescence and the factorial structure of the brief resilience scale (BRS) in Mexican and Chilean University students. Ann Psychol.

[50] Pallant JF, Tennant A (2007). An introduction to the Rasch measurement model: an example using the hospital anxiety and depression scale (HADS). Br J Clin Psychol..

[51] Berg SK, Rasmussen TB, Thrysoee L, Thorup CB, Borregaard B, Christensen AV (2018). Mental health is a risk factor for poor outcomes in cardiac patients: findings from the national DenHeart survey. J Psychosom Res.

[52] Martin CR, Thompson DR, Chan DS (2004). An examination of the psychometric properties of the hospital anxiety and depression scale in Chinese patients with acute coronary syndrome. Psychiatry Res.

