# Supplementary material for: Psychometric properties of the Danish Hospital Anxiety and Depression Scale in patients with cardiac disease: results from the DenHeart survey
Source: Health Qual Life Outcomes. 2020 Jan 7;18:9. doi: 10.1186/s12955-019-1264-0 (PMC6947856; doi:10.1186/s12955-019-1264-0)
Supplement: Supplementary file 1 — Additional file 1: Table S1. Translation Validity Index (TVI) for the Danish translation of Hospital Anxiety and Depression Scale (HADS) [file 12955_2019_1264_MOESM1_ESM.docx]

**Supplementary Table. Translation Validity Index (TVI) for the Danish translation of Hospital Anxiety and Depression Scale (HADS)**

|  | **Range of rating^a^** | **% of score 3 or 4** |
| --- | --- | --- |
| **HADS-A** |  |  |
| Item 1. I feel tense or 'wound up' | 4 | 100% |
| Item 3. I get a sort of frightened feeling as if something awful is about to happen | 1-4 | 60% |
| Item 5. Worrying thoughts go through my mind | 3-4 | 100% |
| Item 7. I can sit at ease and feel relaxed | 3-4 | 100% |
| Item 9. I get a sort of frightened feeling like 'butterflies' in the stomach | 3-4 | 100% |
| Item 11. I feel restless as I have to be on the move | 1-4 | 60% |
| Item 13. I get sudden feelings of panic | 3-4 | 100% |
| **HADS-D** |  |  |
| Item 2. I still enjoy the things I used to enjoy | 3-4 | 100% |
| Item 4. I can laugh and see the funny side of things | 3-4 | 100% |
| Item 6. I feel cheerful | 3-4 | 100% |
| Item 8. I feel as if I am slowed down | 4 | 100% |
| Item 10. I have lost interest in my appearance | 3-4 | 100% |
| Item 12. I look forward with enjoyment to things | 3-4 | 100% |
